# *Mallotus philippensis* Extract‐Infused Locust Bean Gum‐Based Ternary Hydrogel: A Green Fabrication Approach for the Controlled Release of Levofloxacin

**DOI:** 10.1002/mabi.202500266

**Published:** 2025-07-14

**Authors:** Sehrish Jabeen, Atif Islam, Rafi Ullah Khan, Dirk W. Schubert

**Affiliations:** ^1^ Institute of Polymer Materials, Department of Material Science and Engineering, Faculty of Engineering Friedrich‐Alexander University Erlangen‐Nuremberg Martensstrasse 7 Erlangen Bavaria Germany; ^2^ Institute of Polymer and Textile Engineering, Faculty of Chemical and Materials Engineering University of the Punjab Lahore Punjab Pakistan; ^3^ School of Chemistry, Faculty of Science University of the Punjab Lahore Punjab Pakistan; ^4^ Institute of Chemical Engineering and Technology, Faculty of Chemical and Materials Engineering University of the Punjab Lahore Punjab Pakistan

**Keywords:** (3‐Aminopropyl)triethoxysilane, Antibacterial response, Levofloxacin, Lucast bean gum, *Mallotus philippensis* extract

## Abstract

Smart and responsive drug delivery systems are key to next‐generation biomedical therapies, offering precision and targeted action. This study reports the development of a novel biodegradable, pH‐sensitive, and highly swellable hydrogel composed of locust bean gum, polyvinylpyrrolidone, chitosan, and (3‐aminopropyl)triethoxysilane (APTES) as a crosslinker, incorporating *Mallotus philippensis* (MP) extract to enhance bioactivity. Structural, thermal, and morphological properties were characterized by FT‐IR, TGA, and SEM. Swelling behaviour confirmed pH responsiveness, while gel content and biodegradation assays verified stability and degradability. Contact angle and porosity analyses showed favourable surface wettability and porous architecture. Antimicrobial activity demonstrated inhibition of bacterial strains, with cytocompatibility supported by brine shrimp lethality assay. Levofloxacin (LVX) was loaded into hydrogels with and without MP extract, achieving drug encapsulation efficiencies of 89% and 85%, respectively, with a slight decrease attributed to phytoconstituents interactions affecting network density. Drug release profiling at pH 5.5, 6.5, and 7.4 showed sustained release exceeding 80% within 3 h per USP standards. LPC‐3AT and LPC‐3AT‐MP 400 released 87.04% and 94.5% LVX over 180 min in PBS, following a non‐Fickian (anomalous) transport mechanism (diffusion exponent n = 0.62). These findings highlight the hydrogel's promise as an injectable platform for controlled drug delivery and advanced biomedical applications.

## Graphical abstract

A novel bio‐based hydrogel was developed using *Mallotus philippensis* extract, Lucast bean gum, and chitosan for controlled levofloxacin release. The hydrogel exhibited excellent swelling behavior, antimicrobial activity, and pH‐responsive drug release, demonstrating its potential for advanced wound healing and targeted drug delivery applications.

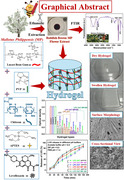

## 1 Introduction

Tomorrow's therapy begins with today's smart systems—targeted, gentle, and enduring in their healing purpose, with precision‐controlled and sustained drug release at their core [1]. Traditional drug administration routes, such as oral or intravenous delivery, often lead to rapid systemic distribution, requiring high doses that can result in toxicity and reduced patient compliance [2, 3].

As an alternative, hydrogels, the hydrophilic, crosslinked polymeric networks capable of retaining large volumes of water, have emerged as promising platforms for localized and sustained drug delivery. Their biocompatibility, tunable porosity, and ability to mimic extracellular matrices make them particularly attractive for applications in tissue engineering [4], wound healing [5] drug delivery, and infection control [6, 7]. Optimizing hydrogel performance requires the deliberate selection of materials to achieve the desired biological activity, stability, and release behaviour. Accordingly, in this study, we designed and fabricated a multifunctional hydrogel with carefully chosen matrix components. Each component plays a specific role in enhancing the overall functionality of the hydrogel, starting with chitosan.

Chitosan, (CS) a natural polysaccharide derived from chitin, is composed of (1→4)‐linked D‐glucosamine and N‐acetyl‐D‐glucosamine units [8]. CS offers excellent biocompatibility [9], biodegradability [10], inherent antimicrobial [11] and anti‐inflammatory [12] properties due to its polycationic nature. The active hydroxyl and amine groups on CS backbone, make it highly functional for multiple applications, including food [13], cosmetics [14], biotechnology [15], and pharmaceuticals [16].

Polyvinylpyrrolidone (PVP), a synthetic, water‐soluble polymer with exceptional hydrophilicity and film‐forming capacity, enhances the mechanical strength, elasticity, structural integrity, and drug‐encapsulation efficiency of hydrogels [17, 18]. The drug release behaviour of metronidazole from chitosan–PVP hydrogels, as observed by Feyissa et al. (2023), supports the potential of these polymers in enhancing drug encapsulation, delivery efficiency, and structural performance [19].

Locust Bean Gum (LBG) is a galactomannan polysaccharide that serves as a non‐toxic, biocompatible natural polymer. When blended with synthetic polymers, LBG can reduce its cytotoxicity and enhance the overall bio‐acceptability of the hydrogel. It forms strong hydrogen bonds with polymers such as chitosan or PVP, resulting in a denser and more stable hydrogel network that improves mechanical strength and gel consistency. Additionally, the combination of LBG with hydrophilic polymers increases the hydrogel's swelling capacity and water retention. LBG also slows down the degradation of the polymer matrix and modulates drug diffusion, thereby enabling sustained or targeted drug release — a crucial feature for controlled drug delivery systems [20, 21]. Matar et al. (2023) also reported similar properties of LBG [22].

(3‐Aminopropyl)triethoxysilane (APTES), a silane‐based crosslinker, was employed to chemically reinforce the 3D network, thereby enhancing the hydrogel's structural integrity and stability under physiological conditions [23, 24].

*Mallotus philippensis* (MP), commonly referred to as the Kamala tree, is a natural source of diverse bioactive compounds including flavonoids, phenolic acids, triterpenoids, and rottlerin [25, 26]. *Mallotus philippensis* (MP) extract is rationally induced in the designed hydrogel system due to its rich phytochemical profile, which aligns well with the goals of creating bioactive, multifunctional, and eco‐friendly hydrogels.

These phytochemicals exhibit well‐documented antibacterial, anti‐inflammatory, antioxidant, antifungal, and wound‐healing activities [27, 28]. MP extract can synergize with chitosan's inherent antibacterial properties, broadening the antimicrobial spectrum and improving inhibition of both Gram‐positive and Gram‐negative bacteria. Phytochemicals in MP increase bacterial membrane permeability and induce oxidative stress, leading to enhanced bacterial cell death. Khajuria et al. (2024) also explored the in vitro antioxidant and antibacterial activities of *Mallotus philippinensis* leaf extract [26].

The presence of natural antioxidants helps in neutralizing reactive oxygen species (ROS), which is critical in chronic wound environments where oxidative stress delays healing. Abbas et al. (2019) reported the role of curcumin extract loaded onto chitosan and PVA hydrogel in reducing the oxidative stress to support wound healing [29].

Being a plant‐derived compound, MP supports the green synthesis approach and improves the overall biocompatibility of the hydrogel. Various plant extracts, including green tea polyphenols [30], aloe vera [31], neem [32], mint leaves [33], and grapefruit peel extract [34], have been extensively reported in the literature for their incorporation into hydrogels to enhance both their properties and therapeutic efficacy.

Levofloxacin, a broad‐spectrum fluoroquinolone antibiotic, is used as a model drug in hydrogels due to its potent antibacterial activity against a wide range of Gram‐positive and Gram‐negative bacterial strains. Its excellent solubility, stability, and tissue penetration enhance its therapeutic efficacy. Incorporating it into hydrogels enables controlled drug release, reduces dosing frequency, provides localized antimicrobial action, and minimizes side effects [35].

The objective of this study is to investigate the combined effect of a nutraceutical plant extract and a pharmaceutical agent on the structural integrity, antimicrobial performance, and drug delivery capabilities of a bioinspired multifunctional hydrogel system.

This study presents a novel, pH‐sensitive, eco‐friendly, highly swellable, ternary hydrogel system **c**omposed of locust bean gum (LBG), PVP/CS, crosslinked using (3‐aminopropyl)triethoxysilane (APTES) infused with *Mallotus philippensis* (MP) extract (at varying concentrations), also loaded with the model antibiotic levofloxacin (LVX). The release profiles of *Mallotus philippensis* (MP) extract and the model drug Levofloxacin (LVX) were studied. Additionally, LVX release from hydrogels with and without MP extract was evaluated at three different pH values (5.5, 6.5, and 7.4), and to the best of our knowledge, no such study has been reported to date.

**FIGURE 1:**
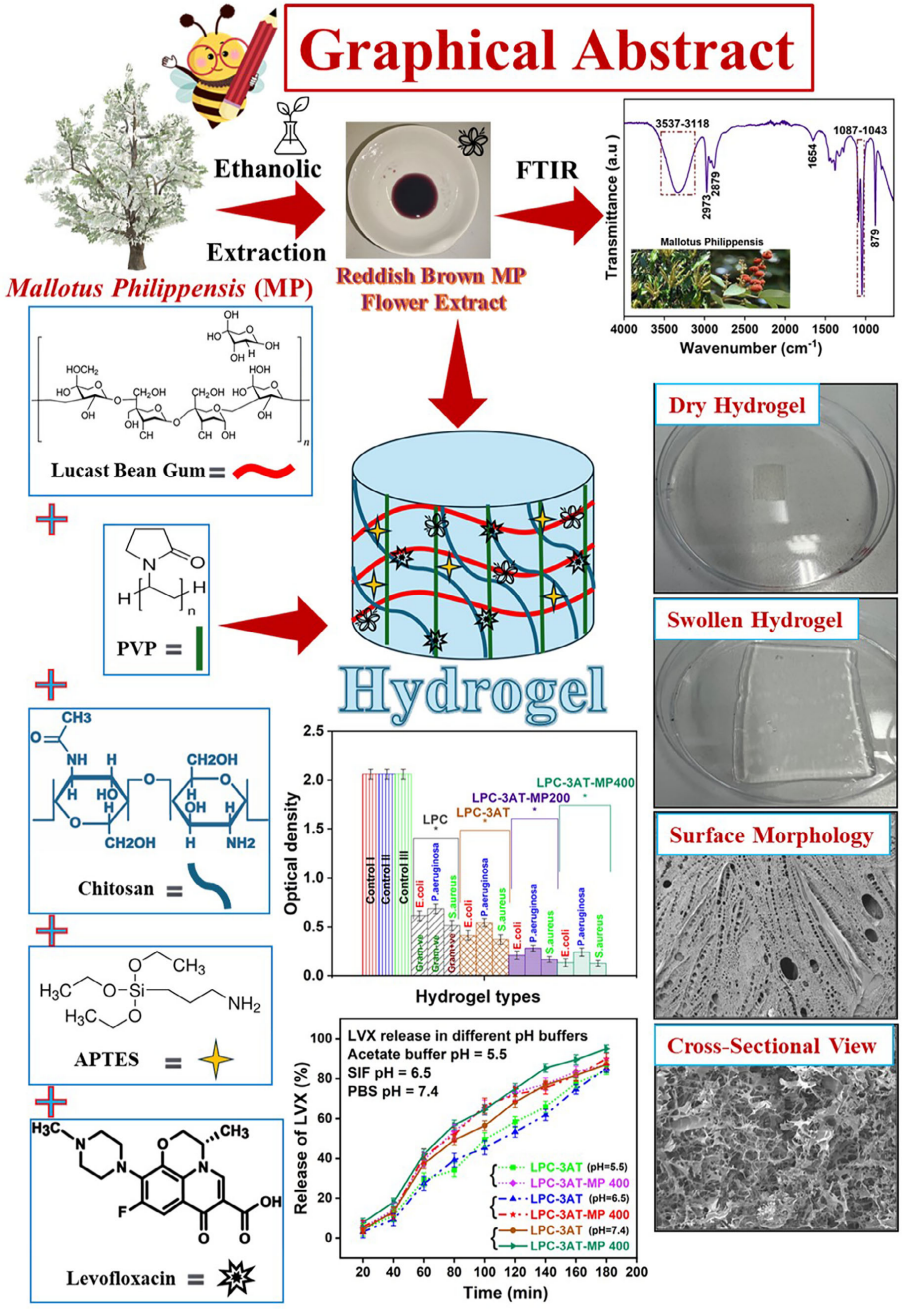
Graphical abstract illustrating a key step, the main compositional elements, and the significant outcomes of the study.

A comprehensive analysis of the hydrogels’ physicochemical properties was conducted. To investigate their structural, thermal, and morphological features, the synthesized hydrogels were subsequently examined using FTIR, TGA, and SEM techniques. Swelling behavior was analyzed in distilled water, different pH buffers, and electrolytes to assess their responsiveness under physiological conditions, while gel content/crosslinking density and in vitro biodegradation were evaluated to determine the stability and degradation profiles. The surface wettability was examined through contact angle measurements, and porosity was assessed to understand the hydrogel's internal structure and drug‐loading capacity. The antimicrobial activity was tested to evaluate its efficacy against both Gram negative and Gram positive bacterial pathogens, and cytocompatibility was assessed through in vitro brine shrimp lethality assay. The release kinetics of *Mallotus philippensis* (MP) extract was also accessed in PBS. The drug encapsulation efficiency (DEE %) and levofloxacin (LVX) release profile were investigated across different physiological conditions—acetate buffer (pH 5.5), simulated intestinal fluid (pH 6.5), and phosphate‐buffered saline (pH 7.4)—along with kinetics to elucidate the controlled release mechanism, highlighting the hydrogel's promise as a localized and sustained drug delivery platform. A visual summary of the study, including a key step, its main compositional elements, and outcomes, is presented as a graphical abstract (Figure 1).

## 2 Experimental Details

### 2.1 Materials

Chitosan (medium molecular weight, deacetylated chitin, Poly(D‐glucosamine), CAS: 9012‐76‐4, Lot No. STBG8451), purchased from Sigma‐Aldrich (USA), with ≥ 95% purity. Locust bean gum (Galactomannan polysaccharide, manno‐galactan as the main component, CAS No. 9000‐40‐2), purchased from Sigma‐Aldrich (USA), with ≥ 95% purity. Polyvinylpyrrolidone (PVP, Mw = 40 000 g/moL) and acetic acid (CH₃COOH, 100% extra pure), purchased from Merck (Germany), with ≥ 99.5% purity. Aminopropyltriethoxysilane (97%), purchased from Sigma‐Aldrich/Merck (Germany), with ≥ 97% purity. Levofloxacin (98.0‐102.0% on anhydrous basis, CAS No. 100986‐85‐4, Lot No. 0000229977), purchased from Sigma Aldrich (USA), with ≥ 98% purity. Ethanolic extract of *Mallotus philippensis* was prepared in the laboratory. Buffer solutions of pH 2, 4, 5.5, 6, 7, 7.4, 8, and 10 were purchased from Sigma‐Aldrich (USA), with ≥ 99% purity. Only analytical grade chemicals were used in this study, without the need for further purification or treatment.

### 2.2 Methodology

#### 2.2.1 Extraction of *Mallotus philippensis* (Kumkum Tree) Extract

The ethanolic extract of *Mallotus philippensis* flowers was prepared using the cold extraction method. The flowers of *Mallotus philippensis* were thoroughly washed with water to remove any dust. The cleaned flowers were first blotted dry using filter paper and then air‐dried in the shade for 3 days. Subsequently, they were dried in an oven at 50°C for 2.5 h and stored in a vacuum desiccator until further use. The completely dried flowers were ground, the powder was sieved to obtain a uniform particle size, and it was stored in an airtight bottle. For extraction, 10 g of this powder was mixed with 50 mL of ethanol in a sample bottle and stored in a dark place at room temperature for 14 days. Finally, all the extract was infused into the ethanol, resulting in a brownish‐tinted liquid that was ready for use [25, 36]. This ethanolic extract is written as MP throughout the article.

#### 2.2.2 Preparation of Hydrogel

Chitosan (0.8 g) was dissolved in 40 mL of 2% acetic acid solution and stirred at 60°C for 3 h. PVP (0.8 g) was added to 40 mL of preheated distilled water at 90°C and stirred until completely dissolved. The two solutions were then mixed and stirred for 1 h at 50°C. LBG (0.4 g) was dispersed in 30 mL of distilled water and magnetically stirred at 50°C until a smooth gel was formed. This gum solution was then combined with the CS/PVP blend and stirred for 1 h at 50°C. This physically crosslinked ternary blend was named LPC. Into this ternary blend (LPC), 200 µL of ethanolic extract of *Mallotus philippensis* (MP) was introduced and stirred under the same conditions. Finally, APTES (100 µL), dissolved in 5 mL of ethanol, was added dropwise into the ternary blend containing the MP extract and stirred for 2 h. The resulting blend was then poured into a petri dish and dried in a vacuum oven (LVO‐2040, Lab Tech, Korea) at 50°C. Using the same procedure, four different hydrogels were prepared. A physically crosslinked ternary blend, serving as the control hydrogel, was named LPC. The other three hydrogels were chemically crosslinked with a consistent concentration of the crosslinker. The hydrogel containing 100 µL of the crosslinker APTES was coded as LPC‐3AT. To study the effect of *Mallotus philippensis* (MP) extract, two concentrations (200 and 400 µL) were used, and the hydrogels were coded as LPC‐3AT‐MP 200 and LPC‐3AT‐MP 400, respectively. To ensure the removal of residual acid, the prepared hydrogels were washed at least three times with deionized water until the washings reached neutral pH. This step effectively eliminated any acidic residue and prevented potential interference with the hydrogel's properties or biological performance. To further ensure complete removal of volatile and acidic remnants, the hydrogels were also subjected to vacuum oven drying at 50°C for 24 h. This additional step aids in the evaporation of any trace residual acid and enhances the structural stability of the hydrogels. The compositions and codes of each hydrogel formulation are listed in Table 1.

**TABLE 1:** Compositions and identification codes used in LPC hydrogel formulations.

Serial No.	Formulation codes	Lucast bean gum (g)	PVP (g)	Chitosan (g)	*Mallotus philippensis* (MP) (µL)	3‐APTES (µL)	Levofloxacin (LVX) (mg)
1	**LPC**	0.4	0.8	0.8	–	–	–
2	**LPC‐3AT**	0.4	0.8	0.8	–	100	50
3	**LPC‐3AT‐MP 200**	0.4	0.8	0.8	200	100	–
4	**LPC‐3AT‐MP 400**	0.4	0.8	0.8	400	100	50

## 3 Characterizations and Physicochemical Analyses

### 3.1 Fourier Transform Infrared Spectroscopy (FTIR)

FTIR spectra were recorded using the ATR technique with a ZnSe crystal on a Shimadzu IR‐Prestige‐21 system. The spectra were scanned over the range of 4000–400 cm⁻¹ with a resolution of 4 cm⁻¹, and each spectrum was averaged over 60 scans per sample.

### 3.2 Scanning Electron Microscopy (SEM)

Scanning electron microscopy (SEM) analysis was conducted to study the surface morphology and cross‐sectional views of the fabricated hydrogels. SEM analysis was performed using AURIGA Cross Beam (Carl Zeiss Microscopy GmbH, Germany). Initially, the gold layer of 7.5 nm was coated on samples with a sputter coater (Q150T Turbo Pumped Sputter Coater, Guelph, ON, CA). SEM images were acquired at various magnifications using a secondary electron detector at an acceleration voltage of 5 kV.

### 3.3 Thermogravimetric Analysis (TGA)

Thermograms were attained on a TA Instrument SDT Q600 model. The TGA analysis was performed in an inert nitrogen atmosphere at a flow rate of 15 mL/min. Samples were heated from ambient temperature to 600°C at a controlled rate of 10°C/min.

### 3.4 Swelling Studies in Various Media

To conduct the swelling studies in distilled water, pH buffers, and ionic solutions, the dried hydrogel samples were cut into small pieces, weighed (25 mg), and placed in a glass vial containing 40 mL of the respective solvent. In water after every 10 min, the excess solution was discarded, and the vials were cleaned with tissue paper to measure the weight of the swollen hydrogel. This practice was exercised at the same time intervals until the equilibrium was achieved.

Whereas, in different pH buffers and ionic solutions the hydrogels were immersed until their respective equilibrium time was first determined in water. To improve the accuracy of the results, the analysis was performed three times to calculate the mean values. Equation (1) was used to calculate the degree of swelling:

(1) $$Swelling\left(\frac{g}{g}\right)=\frac{W_{s}-\;W_{d}}{W_{d}}\;$$

where W_d_ is the weight of the dry hydrogel and W_s_ is the weight of the swollen hydrogel. Buffer solutions with pH values of 2, 4, 6, 7, 8, and 10 were used to investigate the hydrogels' response to pH. Ionic solutions of NaCl and CaCl₂ at various concentrations (0.2, 0.4, 0.6, 0.8, and 1 m) were used to examine the swelling behaviour of hydrogels [8].

### 3.5 Gel Fraction

The gel fraction/Cross‐linking density was determined by immersing pre‐measured hydrogel samples in plenty of distilled water in a beaker for 10 h. After this period, the water was removed, and the hydrogel samples were dried in a vacuum oven at 40°C until a constant weight was achieved. Equation (2) was used to determine the gel fraction:

(2) $$Gel\;fraction\;\left(\%\right)=\frac{M_{1}}{M_{0}}\;\times 100\;$$

where M_1_ is the weight of the crosslinked sample after the oven drying procedure, and M_0_ is the sample's original weight [10].

### 3.6 In Vitro Biodegradation

The in vitro biodegradation of LPC hydrogels was performed in phosphate‐buffered saline (PBS, pH 7.4). Uniform pieces of hydrogels were cut, weighed (W₀), and immersed in 50 mL of PBS in separate beakers. At specific intervals (1, 3, 5, 7, 9, 11, and 13 days), the samples were removed, gently blotted with tissue paper, and dried in a vacuum oven at 50°C until a constant weight (W_t_) was achieved. The dried hydrogels were reweighed, and weight loss (%) was calculated using Equation (3):

(3) $$Biodegradation\;\left(\%\right)=\frac{W_{0}-W_{t}}{W_{0}}\;\times 100$$

where W_0_ is the initial dry weight, and W_t_ is the weight at time t (after drying). The experiment was repeated in triplicate to ensure reliable results [37].

### 3.7 Contact Angle

A Goniometer (KSV NIMA Digidrop) was used to measure the contact angle of a liquid droplet on a solid surface using the sessile drop method. The water droplet was carefully placed on the hydrogel surface, and an image was captured. The surface water contact angle values were determined by analyzing the droplet profile, and the average of the left and right contact angles was calculated. The process was repeated at least 3 points on the surface to ensure accuracy.

### 3.8 Porosity

The solvent displacement method was used to evaluate the porosity of the developed LPCs. A Vernier calliper (Fowler 6/150 mm Pro‐Max Electronic Calliper 54‐200‐777‐1) was used to measure the dimensions of the LPC samples and determine their volume. Pure ethanol was used as the displacement solvent. To allow the ethanol to penetrate the hydrogel pores, pre‐weighed hydrogel films were submerged in 100% ethanol for 24 h. After removing excess ethanol from the surface of the samples, the samples were weighed. The porosity percentage was calculated using the following Equation (4).

(4) $$Porosity\;\left(\%\right)=\frac{\left(M_{2}\;-\;M_{1}\right)}{\left(\rho \;\times \;V\right)}\times 100$$

where M_2_ and M_1_ denote the weights of the hydrogel samples after and before immersion in ethanol, respectively. ρ referred to the density of ethanol, and V represented the volume of the hydrogel samples.

### 3.9 Antimicrobial Profile

The antimicrobial activity of LPCs was evaluated against Gram‐negative bacteria, Escherichia coli (*E.coli*) and Pseudomonas aeruginosa (P. aeruginosa), as well as the Gram‐positive bacterium Staphylococcus aureus (S. aureus). The study was performed using the JIS L 1902:2002 standard test method, which is a validated, reliable, and rapid approach for evaluating bacterial resistance on textile and polymeric materials [10]. The Luria‐Bertani (LB) media was prepared by dissolving 10 g of tryptone, 5 g of yeast extract, and 10 g of NaCl in 800 mL of distilled water, adjusted to pH 7.0. The volume was brought to 1000 mL, and the solution was autoclave‐sterilized at 121°C for 15 min. For testing, hydrogel samples (5 × 5 mm) were added to 20 mL of LB media inoculated with 20 µL of respective culture i.e., *E.coli*. A control group without the hydrogel samples was prepared under identical conditions. The optical density (OD) of the bacterial growth was measured at 600 nm using a spectrophotometer (PerkinElmer, Model Lambda25, USA) at regular intervals to assess antibacterial efficacy. The test was conducted in triplicate to ensure reliability [8].

### 3.10 Cytocompatibility Profile

The brine shrimp lethality assay, a widely used technique for toxicity screening, was employed to examine in vitro cytotoxicity. Brine shrimp eggs were incubated for 48 h at room temperature in a container containing sterile seawater with continuous aeration. After successful hatching, active nauplii were separated by attracting them to a brighter area of the hatching vessel and were used for the test on a deep‐well microliter plate. Saltwater (0.2 mL) was added to each well, and the active nauplii were counted. Three hydrogel samples were introduced into the wells containing the nauplii. The well plate was then stored at room temperature in a dark area. The survival of the nauplii was monitored under a microscope (GXM, XPL33230; GT Vision, Haverhill, UK), and mortality was determined using Equation (5).

(5) $$M\;\left(\%\right)=\frac{A-B-N}{G-N}\;\times 100$$

M = % of dead nauplii after 24 h.

A = Number of dead nauplii after 24 h.

B = Average number of the dead nauplii in the blind samples after 24 h.

N = Number of dead nauplii before starting test.

G = Total number of nauplii.

### 3.11 Release Kinetics of *Mallotus philippensis* (MP) Plant Extract

To evaluate the release behaviour of the MP plant extract, two MP‐loaded hydrogel formulations, LCP‐3AT‐MP 200 and LCP‐3AT‐MP 400, were selected. The hydrogels were weighed and immersed in phosphate‐buffered saline (PBS, pH 7.4) at 37°C. At predetermined time intervals (20, 40, 60, 80, 100, 120, 140, 160, and 180 min), a fixed volume of the release medium was withdrawn and replaced with fresh buffer to maintain constant volume and sink conditions. The concentration of MP extract in the collected samples was quantified using UV–Visible spectrophotometry at λ_max_ = 290 nm. The cumulative percentage release was calculated using Equation (6).

(6) $$\mathrm{Percentage}\;\mathrm{Release}\;\left(\%\right)=\frac{Amount\;Released}{Initial\;Amount\;Loaded}\;\times 100$$

where Amount Released refers to the quantity of the substance that has been released over a given period of time, and Initial Amount Loaded refers to the total amount of the substance that was originally loaded into the system. The release data were fitted to various kinetic models to determine the mechanism of release, categorized as Fickian (n ≤ 0.5), non‐Fickian or anomalous (0.5 < n < 1), or Case II transport (n ≈ 1).

### 3.12 Drug Encapsulation Efficiency (DEE %)

To determine the drug encapsulation efficiency, a known weight of the ITH hydrogel was immersed in phosphate‐buffered saline (PBS, pH 7.4) and kept at room temperature for 24 h to allow drug release. After this period, the mixture was stirred for 15 min to ensure complete extraction of the drug, followed by filtration to separate the hydrogel residue. The drug concentration in the filtrate was measured using a UV–Visible spectrophotometer (Perkin Elmer Lambda 25, USA) at a wavelength of 290 nm. The DEE (%) was calculated using Equation (7).

(7) $$\begin{array}{cc} & Drug\;Encapsulation\;Efficiency\;\left(DEE\right)\left(\%\right)\\ & =\frac{Actual\;Drug\;Content}{Theoretical\;Drug\;Content}\times 100 \end{array}$$

where Actual Drug Content refers to the amount of drug that is actually encapsulated in the formulation, and Theoretical Drug Content refers to the total amount of drug initially used during the formulation process.

### 3.13 Analysis for Controlled Release of Drug

#### 3.13.1 Synthesis of Drug Loaded Hydrogel

Chitosan (0.8 g) was dissolved in 40 mL of 2% acetic acid solution and stirred at 60°C for 3 h. PVP (0.8 g) was dissolved in 40 mL of preheated distilled water at 95°C and stirred until completely dissolved. The two solutions were then mixed and stirred for 1 h at 50°C. LBG (0.4 g) was dispersed in 30 mL of distilled water and magnetically stirred at 50°C until a smooth gel was formed. This gum solution was then combined with the chitosan/PVP blend and stirred for 1 h at 50°C. Into this ternary blend, 400 µL of ethanolic MP extract was introduced and stirred under the same conditions. The drug was added just before the crosslinker. LVX (50 mg) was dissolved in 10 mL of distilled water and slowly introduced into the ternary blend containing the MP extract, followed by stirring for 1 h at 50°C. Finally, APTES (100 µL), dissolved in 5 mL of ethanol, was gradually added to the drug‐containing blend and stirred for 2 h. The resulting blend was then poured into a petri dish and dried in an oven at 50°C. This procedure describes the LVX loading process for LPC‐3AT‐MP 400. For LPC‐3AT (without MP extract), the same procedure was followed, excluding the addition of the MP extract.

#### 3.13.2 Drug Release Profiling at Different pH Conditions

The drug release study was performed using three different pH buffer systems to simulate both physiological and pathological conditions: acetate buffer (pH 5.5), simulated intestinal fluid (SIF, pH 6.5), and phosphate‐buffered saline (PBS, pH 7.4). In each case, 100 mL of freshly prepared buffer solution was transferred into a 1000 mL beaker, and a pre‐weighed drug‐loaded hydrogel sample (2.063 g) was immersed in the medium. The release studies were carried out at 37°C to replicate body temperature. At 20‐min intervals, 5 mL aliquots of the release medium were withdrawn and replaced with an equal volume of fresh buffer to maintain sink conditions. Sampling continued for up to 240 min. The concentration of levofloxacin (LVX) released into the buffer was quantified using a UV–Visible spectrophotometer (Perkin Elmer, Lambda 25, USA) at a wavelength of 290 nm. Buffer‐specific calibration curves were prepared using 100 ppm LVX solutions in each corresponding buffer (pH 5.5, 6.5, and 7.4) to ensure accurate quantification under varying pH conditions.

### 3.14 Statistical Analysis

All experiments were conducted in triplicate, and the results are presented as mean ± standard deviation (SD) using Origin Pro 8 software. Statistical significance was assessed using the Student's t‐test, with p < 0.05 considered significant.

## 4 Results and Discussion

### 4.1 Fourier Transform Infrared Spectroscopy (FTIR)

**FIGURE 2:**
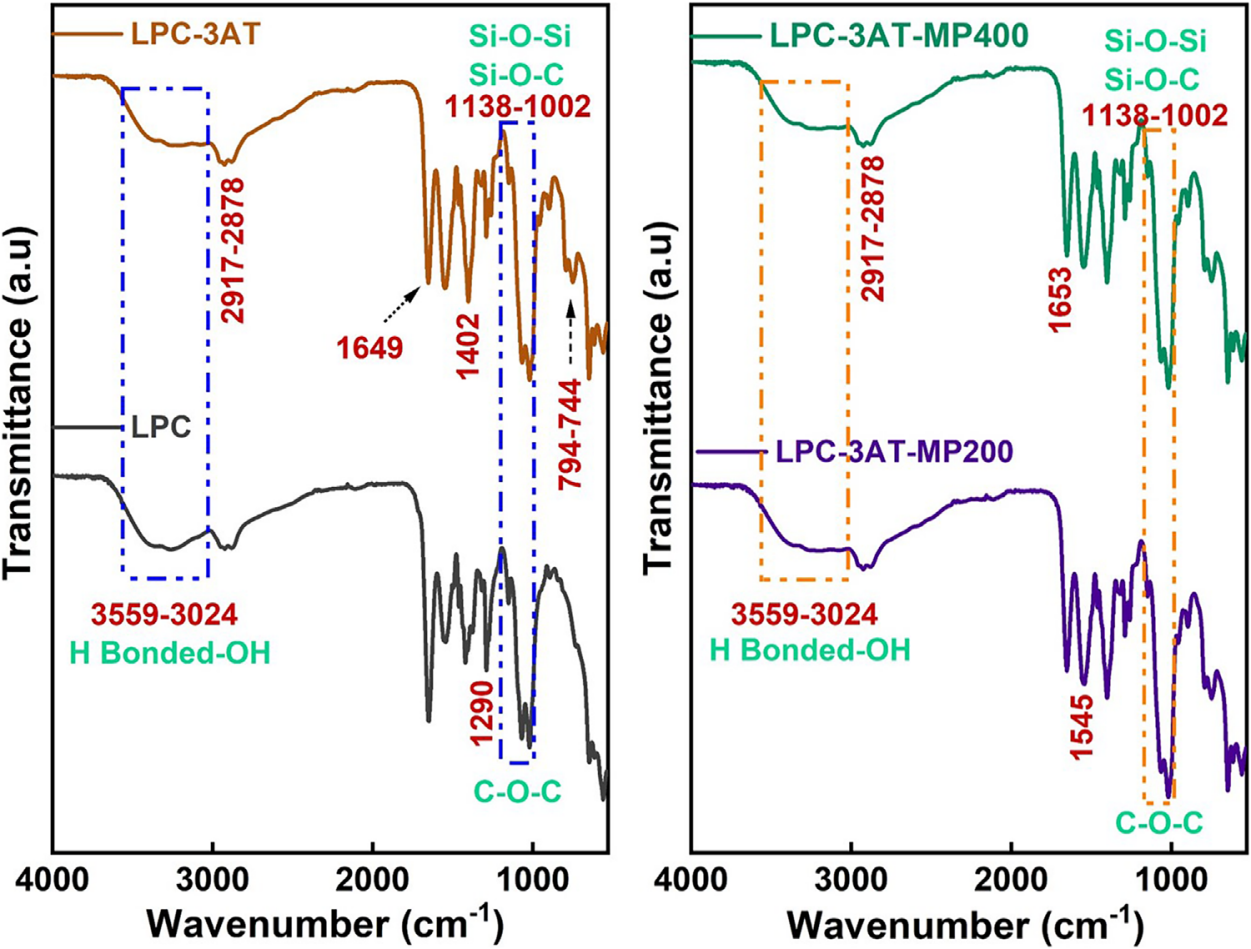
FTIR spectra of LPC (control), LPC‐3AT, LPC‐3AT‐MP 200, and LPC‐3AT‐MP 400 crosslinked hydrogels.

FTIR spectroscopy was conducted to analyze the characteristic absorption peaks of lucast bean gum, PVP, chitosan (LPC), and crosslinked LPC‐3AT, LPC‐3AT‐MP 200, and LPC‐3AT‐MP 400 hydrogels, as shown in Figure 2. A broad band between 3559 and 3024 cm⁻¹ confirmed hydrogen bonding among hydroxyl groups in the polysaccharide backbone. Peaks within 2917–2878 cm⁻¹ were attributed to symmetric and asymmetric C‐H stretching vibrations originating from lucast bean gum, PVP, and chitosan. Stretching vibrations of C‐N bonds in PVP were evident in the range of 1402 cm⁻¹, while carbonyl stretching in the lactam group of PVP was observed at 1649 cm⁻¹. Characteristic amide bands were identified at 1653 cm⁻¹ (amide l), 1545 cm⁻¹ (amide II), 1290 cm⁻¹, (amide III,) and 650 cm⁻¹ (amide V). The pyranose ring, observed at 894 cm⁻¹, confirmed the presence of lucast bean gum and chitosan in the hydrogels. Peaks between 1138–1002 cm⁻¹ represented C─O─C and C─O stretching vibrations, related to glycosidic linkages and sugar rings in lucast bean gum and chitosan. Additionally, the same region revealed Si─O─Si and Si─O─C bonds, indicating successful crosslinking via silane, as supported by previous studies [38]. The 794–744 cm⁻¹ peak in the FTIR spectrum of the hydrogel containing ethanolic MP extract was most likely attributed to aromatic compounds found in the plant extract, particularly flavonoids, phenolic acids, or tannins, which possessed aromatic ring structures. These compounds exhibited characteristic out‐of‐plane C‐H bending vibrations in this region, contributing to the observed peak [6, 8].

### 4.2 Scanning Electron Microscopy (SEM)

**FIGURE 3:**
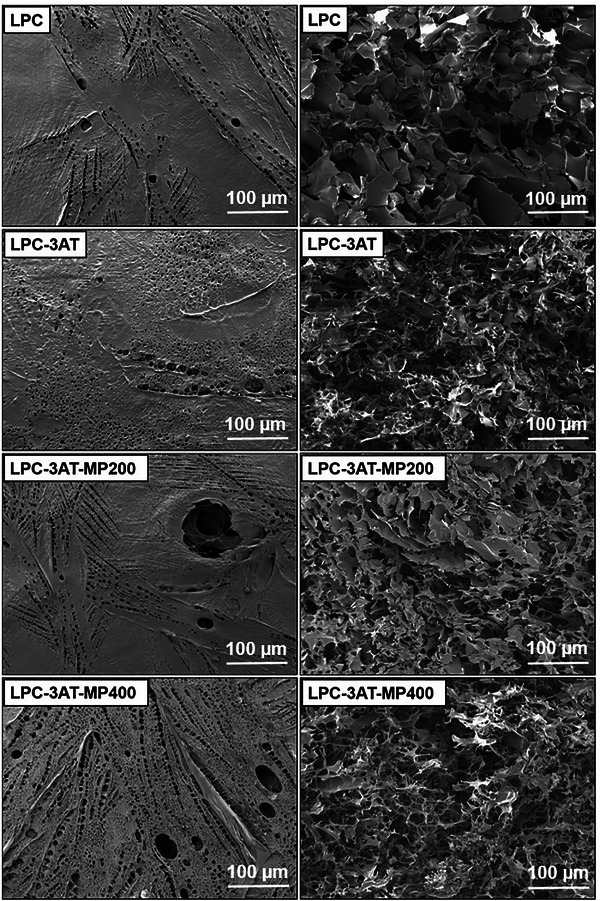
SEM micrographs of LPCs: (Column I) surface morphologies, and (Column II) corresponding cross‐sectional views, Scale bar = 100 µm.

The surface morphologies and cross‐sectional views of fabricated LPCs are shown in Figure 3. The outermost surface of LPC appeared plain and smooth, with numerous visible pores of varying sizes. In contrast, the cross‐sectional view revealed dense, thin sheet‐like aggregates with visible larger pores. After the addition of the crosslinker in LPC‐3AT, the number of pores increased but their size decreased, and the hydrogel surface became uneven, showing noticeable depressions. The internal structure displayed inhomogeneity, as the sheet‐like aggregates transformed into a compact, porous network, though not a finer interconnected one. In LPC‐3AT‐MP 200, the surface appeared relatively uniform and featured abundant micro and macro pores. The layered hydrogel structure was clearly visible, and the inner layers also exhibited porosity. The cross‐sectional view revealed thin, well interconnected porous sheets. With the addition of ethanolic MP extract in LPC‐3AT‐MP 400, the top surface appeared fully covered with abundant nano, micro, and macro‐pores, leaving no plane area visible. The cross‐sectional view revealed that the thin sheet‐like structures transformed into thread‐like formations, which are three‐dimensionally interconnected with increased porosity [23].

### 4.3 Thermogravimetric Analysis (TGA)

**FIGURE 4:**
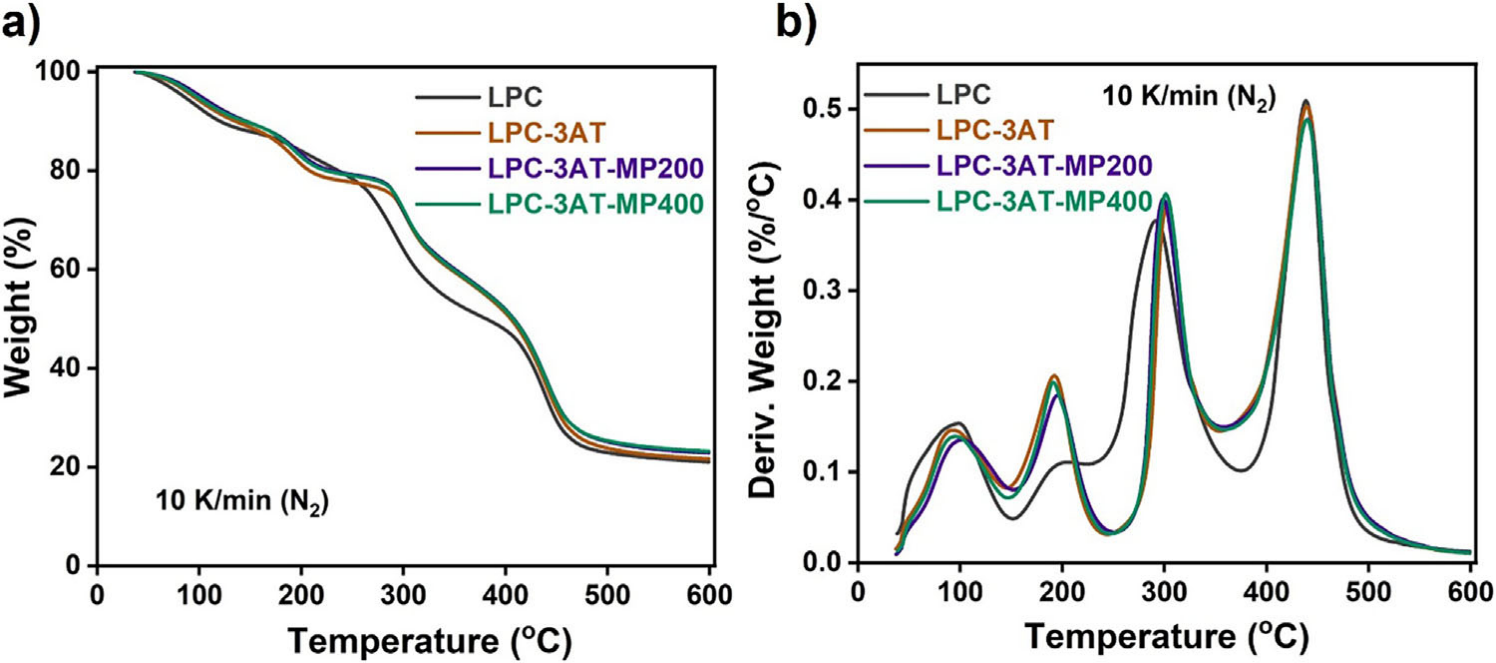
a) TGA Thermograms of LPCs. b) Derivative of TGA.

The thermograms and derivative weight plots of LPC, LPC‐3AT, LPC‐3AT‐MP 200, and LPC‐3AT‐MP 400, shown in Figure 4a,b, were analyzed to examine the influence of the crosslinker and MP extract concentrations on the thermal stability of synthesized LPCs. All the LPCs exhibited a semilinear reduction in total weight during weight loss. The TG thermograms of control hydrogel (LPC) displayed three stages of degradation whereas the three of crosslinked hydrogels showed four stages of decomposition. The DrTG curves also revealed four prominent peak temperatures (Tp's), confirming degradation in four steps: two broad peaks for regions I and II, and two sharp peaks representing major degradation in regions III and IV, as shown in Figure 4b. In the initial step, weight loss was due to evaporation, moisture loss, and the elimination of free water, released up to 100°C. The second step involved the removal of physisorbed water, bound water, and freezing bound water, which were released between 100°C and 200°C. The third and fourth stages marked the major degradation phases: onset (breakdown of secondary forces, functional groups detachment, and polymer interactions) and offset (scission of backbone C–C chains). For LPC, LPC‐3AT, LPC‐3AT‐MP 200, and LPC‐3AT‐MP 400, onset degradation temperatures were 289°C, 290°C, 298°C, and 301°C, while offset temperatures were 437°C, 439°C, 440°C, and 441°C, respectively, as also confirmed by derivative weight loss curves. The 10% weight loss observed at 111°C for LPC, 130°C for LPC‐3AT, 135°C for LPC‐3AT‐MP 200, and 144°C for LPC‐3AT‐MP 400. Similarly, 70% weight loss was recorded at 446°C for LPC, 454°C for LPC‐3AT, 455°C for LPC‐3AT‐MP 200, and 458°C for LPC‐3AT‐MP 400. Residual weights at 600°C were 21.02% for LPC, 21.65% for LPC‐3AT, 22.86% for LPC‐3AT‐MP 200, and 23.18% for LPC‐3AT‐MP 400. The addition of crosslinker and MP extract enhanced thermal stability, with LPC‐3AT‐MP 400 being the most stable [8, 17].

### 4.4 Swelling Studies

#### 4.4.1 Swelling in Water

**FIGURE 5:**
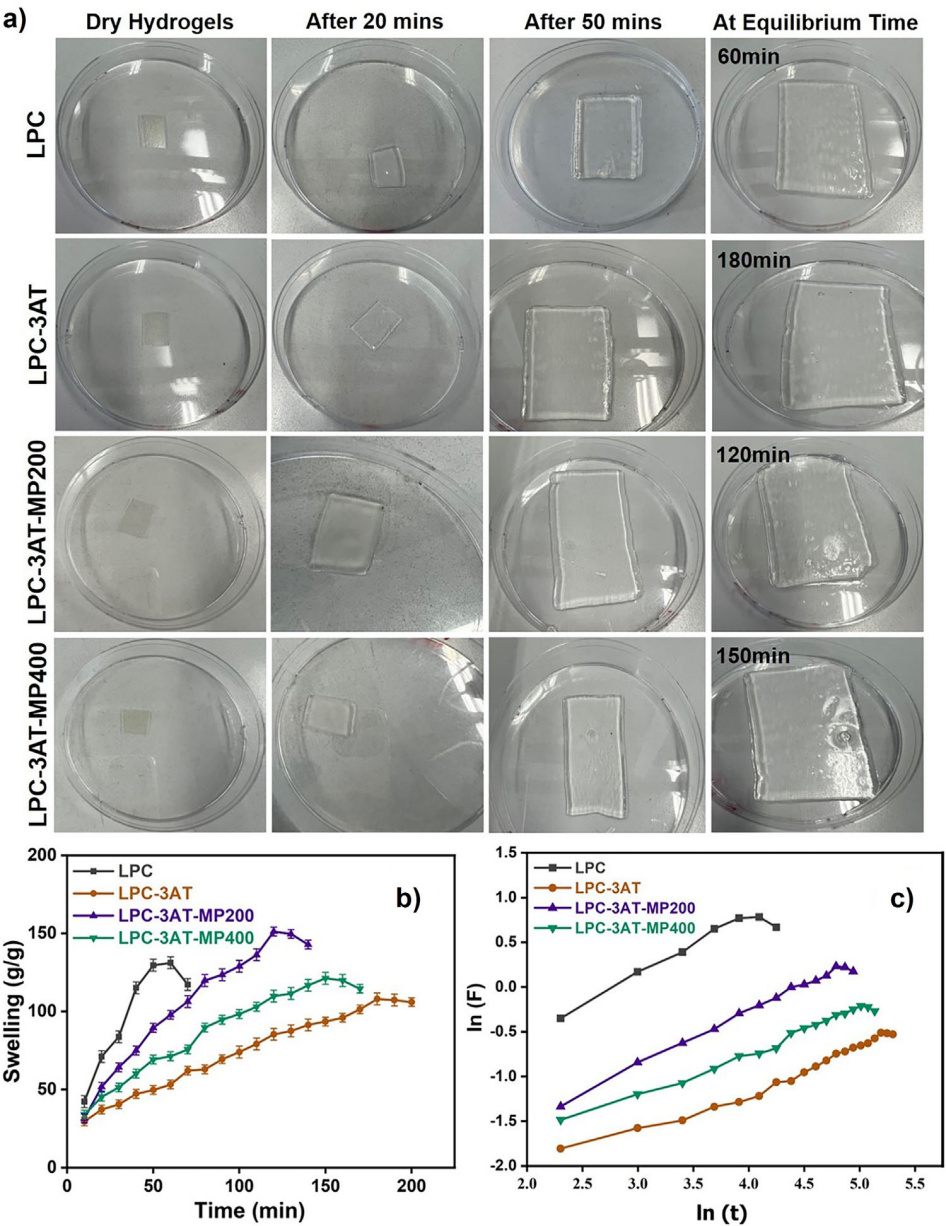
a) The transformation from the un‐swollen to swollen state was tracked visually using time‐lapse photographs until the hydrogels reached equilibrium. b) Hydrogel weight measurements (conducted in triplicate) over time in distilled water at room temperature, along with a plot of swelling (g/g) versus time (min). c) Swelling kinetics determined through a plot of ln(F) versus ln(t).

The diffusion process, polymer group ionization, hydrogen bonding within and between molecules, and the affinity of polymer chains for one another were taken into account to check the swelling of the prepared hydrogels. The transformation from the un‐swollen to swollen state was visually tracked using time‐lapse photographs until the hydrogels reached their equilibrium time. The swelling behaviour in distilled water was measured and plotted as swelling (g/g) versus time (min), as shown in Figure 5a,b. A steady increase in swelling over time was noticed in all the LPC's. In hydrogel LPC‐3AT with addition of crosslinker swelling decreased but the overall equilibrium swelling time was increased. The equilibrium swelling times were revealed to be, 60,180,120, and 150 min for LPC, LPC‐3AT, LPC‐3AT‐MP 200, and LPC‐3AT‐MP 400, respectively.

The physically crosslinked control (LPC) hydrogel exhibited the highest swelling (131.19 g/g) at 60 min because of its less dense structure and larger pore size. LPC‐3AT showed an abrupt decrease in swelling, with a swelling of 108 g/g until its equilibrium swelling time of 180 min. Due to crosslinking, the polymer chains became entangled, forming a more complex and dense interlinked structure that hindered solvent diffusion. However, LPC‐3AT ultimately demonstrated better stability than all other LPCs.

After the addition of ethanolic MP extract, its bioactive components might have disrupted the gel network, facilitating swelling. As a result, LPC‐3AT‐MP 200 showed greater swelling of 151 g/g at 120 min.

With a further increase in MP concentration in LPC‐3AT‐MP 400, the higher polyphenol content led to stronger intermolecular forces between MP and LPC, resulting in a more stable hydrogel structure. Consequently, the swelling decreased to 121.27 g/g, while the equilibrium time increased to 150 min. Similar swelling results were also reported by Jia et al. (2024) using P. suffruticosa Andr. leaf extract (PLE) with chitosan [6, 10]. Swelling studies were performed until each hydrogel reached its respective equilibrium swelling time. Beyond this point, the hydrogel structure became unstable, leading to mechanical failure such as cracking, tearing, or fragmentation. This resulted in a rapid, non‐ideal, and uncontrolled release behaviour, as indicated by the deviation from the linear swelling trend observed in Figure 5a.

#### 4.4.2 Water Absorption Kinetics

Water diffusion in a hydrogel matrix refers to the movement of water molecules through the polymer network of the hydrogel. The swelling kinetics of LPCs are illustrated in Figure 5c, and the relationship between lnt/lnF is plotted using the swelling data in water. The mechanism of water diffusion was determined by Equation (8).

(8) $$F=k\;\cdot \;t^{n}$$

where *F* represents the fractional swelling, defined as the ratio of swelling at equilibrium time (*W_eq_*) to swelling at time *t* (*W_t_*). The parameter *k* denotes the swelling rate constant, and *n* is the swelling exponent. The values of *k* and *n* were determined based on the swelling behavior of hydrogels immersed in distilled water. The exponent *n* provides insights into the solvent transport mechanism within the hydrogel matrix.

A Fickian transport mechanism is characterized by an n value of less than 0.5, while a non‐Fickian transport mechanism occurs when n is greater than 0.5 but less than 1. The diffusion parameters presented in Table 2 indicate that LPC‐3AT‐MP 200 exhibited an n value of 0.5, suggesting a Fickian diffusion mechanism or a borderline case. In contrast, LPC, LPC‐3AT, and LPC‐3AT‐MP 400 showed an n value greater than 0.5, indicating a non‐Fickian diffusion mechanism [7, 39].

**TABLE 2:** Diffusion parameters of the LPC hydrogel formulations.

Parameters	LPC	LPC ‐3AT	LPC‐3AT‐MP 200	LPC‐3AT‐MP 400
**n**	0.58	0.64	0.5	0.607
**Intercept**	−1.60	−3.03185	−2.64274	−2.63516
**K**	0.2018	0.0482	0.0711	0.07170
**Regression (R %)**	92	97	99	98
**Gel‐Fraction (%)**	65.59	87.67	83.24	85.39

#### 4.4.3 Swelling in Different pH Buffers

**FIGURE 6:**
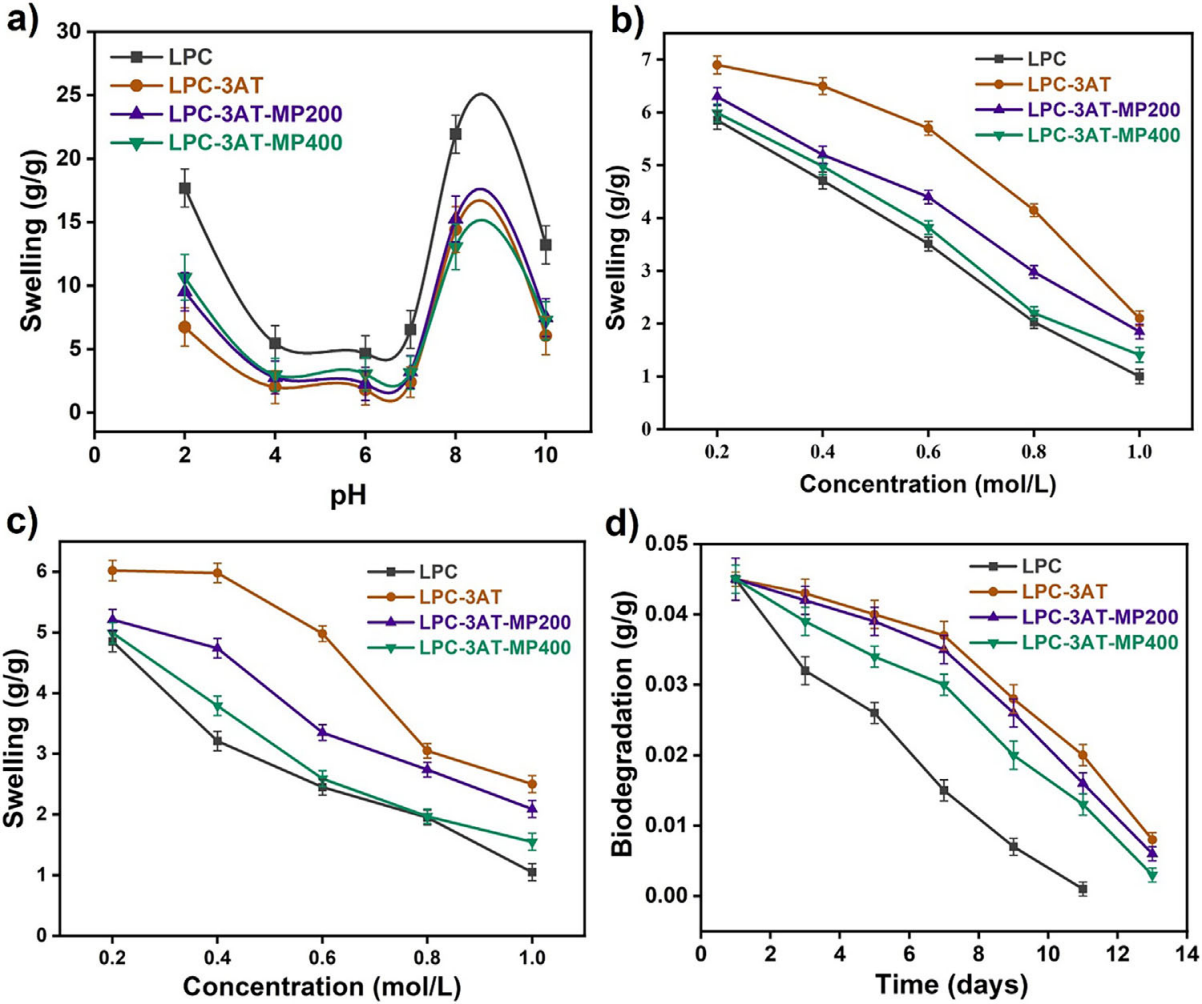
a) Swelling behaviour of LPCs in different pH buffer solutions, b) swelling response in NaCl c) swelling response in CaCl_2_ d) biodegradation of LPCs in PBS.

The swelling behaviour of hydrogels LPC, LPC‐3AT, LPC‐3AT‐MP 200, and LPC‐3AT‐MP 400 as a function of pH is shown in Figure 6a. Swelling was measured as grams of water absorbed per gram of hydrogel (g/g) at different pH values. The LPC hydrogel exhibited the highest swelling at both pH 2 and pH 8‐10. The other hydrogels (LPC‐3AT, LPC‐3AT‐MP 200, and LPC‐3AT‐MP 400) followed a similar trend but with lower swelling capacity than LPC. All hydrogels showed minimal swelling in the pH range of 4–7, indicating reduced water absorption under neutral and slightly acidic conditions. The highest swelling at pH 2 was observed due to the protonation of amine (─NH₃⁺) groups of chitosan in acidic conditions, resulting in electrostatic repulsion between the polymer chains. This repulsion led to network expansion and enhanced swelling. At pH 8–10, the chitosan and PVP functional groups deprotonated, leading to charge repulsion, which increased the hydrogel's ability to absorb water and expand. However, in the pH range of 4–7, ionization of the functional groups is minimal, resulting in a more compact polymer structure with fewer electrostatic repulsions, thereby reducing the swelling capacity [37].

#### 4.4.4 Swelling in Ionic Solutions

NaCl and CaCl₂ were chosen as model electrolytes to represent the diverse range of electrolytes found in the human body's complex composition. These electrolytes consist of monovalent Na⁺ and divalent Ca^2^⁺ ions, which differ in their charge‐to‐size ratio. In ionic environments, both the type and concentration of salts influence the swelling behavior of hydrogels. To evaluate the swelling behavior of the synthesized LPCs, a series of NaCl and CaCl₂ solutions with varying molar concentrations were used, as shown in Figure 6b,c. Although both salts share the same anion (Cl⁻), their cations (Na⁺ and Ca^2^⁺) differ, significantly affecting hydrogel swelling. At lower salt concentrations, complex formation occurred, leading to pore expansion within the hydrogel. However, as salt concentration increased, the osmotic pressure difference between the external solution and the hydrogel decreased, resulting in reduced swelling. The effect was more pronounced in the presence of Ca^2^⁺, as the larger divalent ions hindered solvent penetration, further limiting hydrogel swelling. This one is good but now write it in your own words with change sentences structure [7, 37].

### 4.5 Gel Fraction

The gel fraction represents the proportion of the hydrogel that remained insoluble (i.e., the crosslinked network) after washing away the soluble components. The gel fraction values for the LPCs, as presented in Table 2, provided a qualitative measure of the extent of crosslinking and network formation. A higher gel fraction percentage indicated a reduction in the swelling capability of the hydrogel due to the increased density of formed crosslinks, which reflected a greater degree of covalent bonding. The physically crosslinked control hydrogel (LPC) exhibited the lowest gel fraction (65.59%), indicating a lower degree of crosslinking and structural stability due to the absence of a chemical crosslinker. In all crosslinked samples, the concentration of the crosslinker was kept constant, ensuring that variations in gel fraction were primarily influenced by the addition of MP extract. Among the crosslinked hydrogels, LPC‐3AT, without MP extract, showed the highest gel fraction (87.67%), demonstrating efficient network formation in the presence of the crosslinker. The introduction of MP extract caused changes in the gel fraction. LPC‐3AT‐MP200 had a reduced gel fraction (83.24%) compared to LPC‐3AT, likely due to partial interference of MP extract components with the crosslinking process. In contrast, LPC‐3AT‐MP400 exhibited a slight increase in gel fraction (85.39%) compared to LPC‐3AT‐MP200, possibly due to the stabilization of the network through secondary interactions, such as hydrogen bonding.

Although direct mechanical testing was not performed, the gel fraction values served as a qualitative indicator of mechanical strength. Hydrogels with higher gel fractions (LPC‐3AT and LPC‐3AT‐MP400) exhibited reduced swelling and enhanced structural integrity, implying stronger and more compact networks. These characteristics inherently slowed down drug diffusion, thereby contributing to a more sustained release profile. Thus, the gel fraction data not only reflected crosslinking efficiency but also provided indirect insight into the gel's mechanical robustness and its impact on drug release kinetics. Andlib et al. (2025) also identified a similar trend in the gel fraction and crosslinking density of rattan jot plant extract‐loaded hydrogels [37].

The gel fraction results correlated well with the swelling trends. Hydrogels with higher gel fractions (LPC‐3AT and LPC‐3AT‐MP400) exhibited lower swelling capacities but greater stability, while those with lower gel fractions (LPC and LPC‐3AT‐MP200) demonstrated higher swelling and faster degradation. This confirmed that gel fraction served as a reliable indicator of both water absorption capacity and hydrogel durability, as well as a predictor of controlled drug release behaviour [10].

### 4.6 Biodegradation

Biodegradation is an essential attribute of hydrogels designed for drug delivery. These hydrogels ensured structural stability for a specific period, enabling the controlled breakdown of the hydrogel after the drug had been completely delivered to the intended location. The in vitro biodegradation of LPCs was evaluated in PBS for 14 days, and the results are presented in Figure 6d. The extent of degradation observed in LPCs was as follows: LPC = 97.8%, LPC‐3AT = 83.3%, LPC‐3AT‐MP 200 = 87.2%, and LPC‐3AT‐MP 400 = 91.4%. The fabricated LPCs are primarily composed of two polysaccharides: locust bean gum and chitosan. Degradation occurred due to the hydrolysis of the glycosidic bonds present in these polysaccharides. The uncrosslinked hydrogel LPC showed faster degradation after day 5, with 97.8% degradation observed by day 11, compared to the crosslinked hydrogel LPC‐3AT, which exhibited faster degradation after day 7 but degraded only up to 83.3% by day 13. The effect of ethanolic MP extract on in vitro biodegradation was estimated while keeping the crosslinker concentration constant. The in vitro biodegradation profile showed a divergent trend from the swelling phenomenon. The hydrogel LPC‐3AT‐MP400, containing a higher concentration of MP extract, exhibited reduced swelling and a higher gel fraction but surprisingly, degraded faster than LPC‐3AT‐MP 200, and vice versa. The ethanolic MP extract contains various hydrophobic compounds (such as phenolics, flavonoids, and alkaloids), which introduce weaker interactions, such as secondary forces and hydrogen bonds. These compounds modify the accessibility of crosslinks to water and create localized microdomains within the polymer network, making the hydrogel more susceptible to enzymatic or hydrolytic degradation. Consequently, the structural integrity of the hydrogels is compromised in PBS, leading to faster degradation [7].

### 4.7 Contact Angle

**FIGURE 7:**
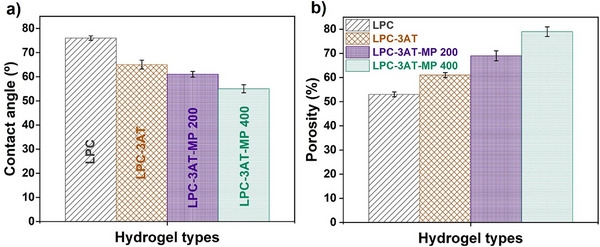
a) Contact angle (°) measurements indicating the surface hydrophilicity of the fabricated LPC hydrogels. b) Percentage porosity of the corresponding hydrogels. The samples presented are LPC, LPC‐3AT, LPC‐3AT‐MP 200, and LPC‐3AT‐MP 400. Data are expressed as mean ± standard deviation (n = 3), and differences were considered statistically significant at p < 0.05.

The contact angle of the LPC hydrogels was investigated as a function of MP content, as shown in Figure 7a and providing valuable insights into surface wettability and hydrophilicity—key factors influencing drug encapsulation efficiency within hydrogel matrices. The LPC control exhibited the highest contact angle (76°), while the values progressively decreased with increasing MP content: 65° for LPC‐3AT, 61° for LPC‐3AT‐MP 200, and 55° for LPC‐3AT‐MP 400. This trend indicates that the incorporation of MP extract effectively enhances the hydrophilic nature of the hydrogels. Since contact angles between 0° and 90° reflect hydrophilic surfaces, all formulations remained within this range, confirming the inherently hydrophilic character of LPC‐based hydrogels. The decreasing contact angle with higher MP concentrations suggests improved surface wettability, allowing water to spread more readily across the hydrogel surface. This enhancement can be attributed to increased porosity and surface roughness introduced by MP extract and APTES, which facilitate stronger water–surface interactions. As porosity increases, the resulting surface roughness enhances the capillary action and absorption potential of the hydrogel, thereby further reducing the contact angle. These findings are consistent with previously reported literature and highlight how structural modifications at the micro‐level through MP loading translate into macroscopic improvements in surface behavior. This improved hydrophilicity not only supports better drug loading and release but also broadens the application potential of these hydrogels in biomedical fields such as wound dressings, drug delivery systems, and bioactive coatings [37, 40].

### 4.8 Porosity

Porosity is a crucial factor in drug release applications, as it enhances the hydrogel's absorption capacity and ensures the even distribution of medication throughout the matrix. All prepared LPCs were evaluated for porosity, as shown in Figure 7b. The crosslinker concentration and the addition of ethanolic MP extract both contributed to an increase in pore volume. The crosslinker APTES plays a key role in the initial rise in porosity by facilitating the formation of interconnected channels through new covalent and hydrogen bonds. This leads to the development of a dense, well‐interconnected network, as further supported by SEM results. Additionally, the incorporation of ethanolic MP extract enhances hydrogel network formation by facilitating additional physical interactions with the polymer chains. When APTES and MP extract are used together, porosity increases from 67% to 75%, demonstrating their synergistic effect on structural enhancement. Notably, there is an indirect relationship between porosity and contact angle: as porosity increases, surface roughness also tends to increase. This enhanced roughness facilitates water penetration into the hydrogel structure, making the material more hydrophilic. Consequently, higher porosity is generally associated with a lower contact angle, reflecting improved wettability of the hydrogel surface [19, 37, 41].

### 4.9 Antimicrobial Studies

**FIGURE 8:**
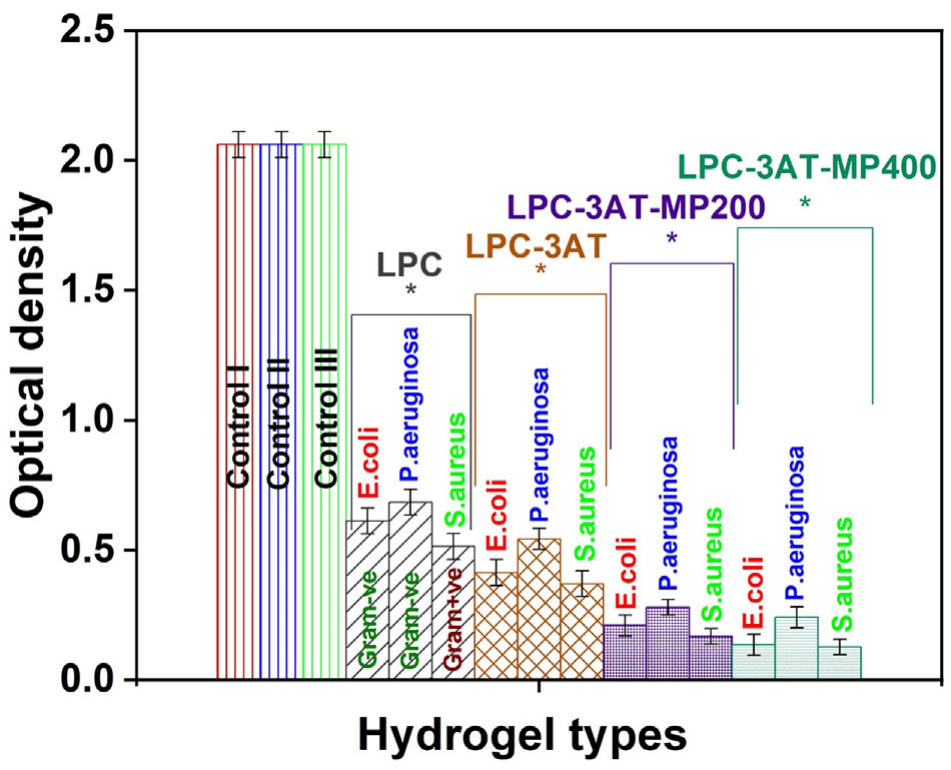
Antimicrobial activity of LPC hydrogels against Gram‐negative *Escherichia coli* (*E. coli*) and *Pseudomonas aeruginosa* (*P. aeruginosa*), and Gram‐positive *Staphylococcus aureus* (*S. aureus*), assessed using sterile Luria‐Bertani (LB) medium.

The antimicrobial activity of LPCs was assessed against both Gram‐negative bacteria (Escherichia coli and Pseudomonas aeruginosa) and Gram‐positive Staphylococcus aureus using sterile Luria‐Bertani (LB) medium. The antibacterial effect was quantified by measuring the optical density (OD) at 600 nm, where lower absorbance values indicate higher bacterial inhibition. The corresponding results are presented in Figure 8. The reference control exhibited the highest absorbance (2.061 ± 0.05), confirming uninhibited bacterial proliferation. In contrast, the experimental control (LPC) hydrogel significantly reduced bacterial growth with absorbance values of 0.612 ± 0.05 (E. coli), 0.684 ± 0.05 (P. aeruginosa), and 0.514 ± 0.05 (S. aureus). This reduction is primarily attributed to chitosan's inherent antimicrobial activity, which arises from its cationic nature that facilitates electrostatic interaction with negatively charged bacterial cell membranes. These interactions compromise membrane integrity, disrupt metabolic functions, and can even hinder transcriptional activity, ultimately leading to bacterial death [42, 43].

Interestingly, although both E. coli and P. aeruginosa are Gram‐negative, the hydrogel showed stronger inhibition against E. coli. This difference can be explained by the intrinsic resistance mechanisms of P. aeruginosa, which possesses a more robust outer membrane, efficient efflux pumps, and an ability to form protective biofilms, all of which reduce the effectiveness of antimicrobial agents, including chitosan‐based systems. On the other hand, S. aureus, a Gram‐positive bacterium, showed the greatest susceptibility, with even lower OD values than E. coli. This heightened sensitivity is due to the absence of an outer membrane in Gram‐positive bacteria, allowing chitosan direct access to the thick, peptidoglycan‐rich cell wall. The strong electrostatic interactions between chitosan and teichoic acids in the cell wall promote rapid membrane destabilization and cellular leakage, leading to higher antimicrobial efficacy [44].

Crosslinking the hydrogel matrix with APTES (LPC‐3AT) further improved antibacterial performance (OD: 0.413 ± 0.05) by enhancing the stability and structural integrity of the hydrogel. This modification likely facilitated better surface contact with bacterial cells and more sustained release of antimicrobial components. The incorporation ofMP plant extract amplified the antimicrobial effects. The LPC‐3AT‐MP 200 formulation exhibited OD values of 0.21 (E. coli), 0.28 (P. aeruginosa), and 0.168 (S. aureus), while LPC‐3AT‐MP 400 achieved the most potent response with 0.13 (E. coli), 0.241 (P. aeruginosa), and 0.127 (S. aureus). These results suggest that the bioactive phytochemicals in MP extract**—**such as flavonoids, tannins, and phenolic acids—acted synergistically with chitosan to disrupt bacterial membranes, induce oxidative stress, and interfere with vital cellular processes. Overall, the progressive decline in absorbance with each structural modification validates the rational design of the LPC hydrogels. The optimized LPC‐3AT‐MP 400 composition, exhibiting the lowest OD values across all tested strains, demonstrates exceptional antibacterial activity, particularly against S. aureus, and stands out as a promising candidate for applications in wound healing, biomedical coatings, and infection‐preventive drug delivery systems [6, 11].

### 4.10 Cytotoxicity Analysis

Cytotoxicity analysis is a key method for evaluating the biocompatibility of hydrogels and their potential effects on living tissues. In this study, in vitro cytotoxicity was assessed using the brine shrimp lethality bioassay, following a previously established and validated protocol (Banti et al., 2021) [45]. As presented in Table 3, all hydrogel formulations exhibited low mortality rates in *Artemia salina* nauplii, ranging from 1.62% for LPC to 1.37% for LPC‐3AT‐MP 400. These low values indicate minimal cytotoxicity and highlight the overall safety of the synthesized hydrogels. The components used—chitosan (CS), polyvinylpyrrolidone (PVP), locust bean gum (LBG), APTES, are widely reported for their biocompatibility and are commonly employed in biomedical applications. Gull et al. (2021) provided evidence of the cytotoxic effects of chitosan and PVP hydrogels using the brine shrimp lethality bioassay [10]. Interestingly, formulations containing MP extract exhibited slightly reduced mortality compared to the base formulation. This may be attributed to the extract's known antioxidant and antimicrobial properties, which can contribute to maintaining a favorable and sterile test environment. Similar properties of MP ethanolic extract were also reported by Ali et al. (2024) [25]. Additionally, slight mortality in all groups may result from physical obstruction due to the formation of dense hydrogel layers on nauplii gills, which can impair oxygen absorption—a phenomenon occasionally noted in hydrogel toxicity studies. Such physical obstruction was also noted by Andlib et al. (2025) in hydrogels loaded with ethanolic RJ plant extract against Artemia salina nauplii [37]. Overall, the low and decreasing mortality across formulations confirms the cytocompatibility of the developed hydrogels and supports their potential for safe application in drug delivery systems, while minimizing adverse biological effects.

**TABLE 3:** Percentage mortality of fabricated LPC hydrogels determined using the brine shrimp lethality bioassay for in vitro cytotoxicity analysis.

Types of hydrogels	Mortality (%)
LPC	1.62
LPC ‐3AT	1.46
LPC‐3AT‐MP 200	1.41
LPC‐3AT‐MP 400	1.37

### 4.11 Release Kinetics of MP Plant Extract

**FIGURE 9:**
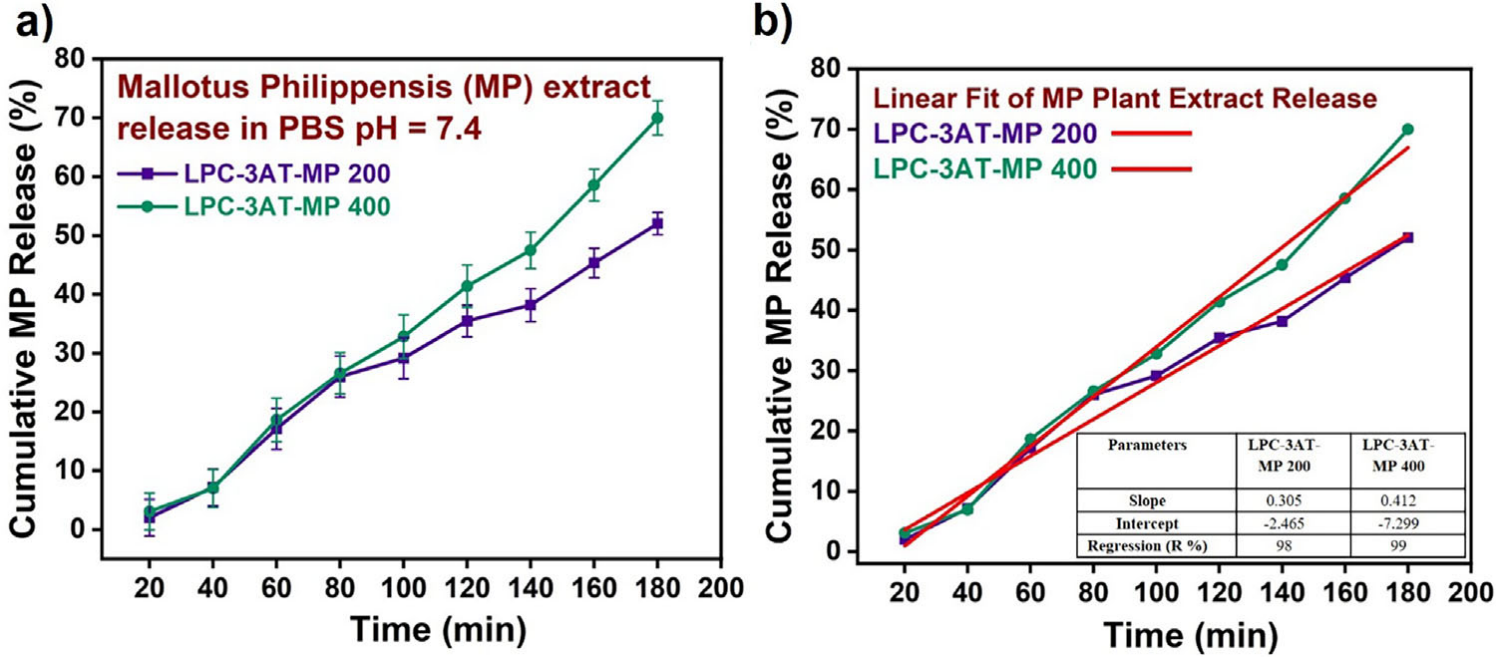
a) Cumulative release (%) of *Mallotus philippensis* (MP) from MP‐loaded hydrogels: LPC‐3AT‐MP200 and LPC‐3AT‐MP400 in PBS (pH 7.4). b) Linear fit applied to the cumulative MP release (%) profiles of LPC‐3AT‐MP200 and LPC‐3AT‐MP400. The corresponding slope, intercept, and regression coefficient (R^2^) values are presented in the table inserted within panel (b).

The release of MP plant extract from the hydrogels was analyzed at physiological pH (7.4) using phosphate‐buffered saline (PBS), and the results are presented in Figure 9a. Among the applied kinetic models, the best fit was observed with the zero‐order model, as indicated by high correlation coefficients (R % = 98 for LPC‐3AT‐MP 200 and 99 for LPC‐3AT‐MP 400) as shown in Figure 9b. Zero‐order kinetics implies that the release of MP occurred at a constant rate, independent of its concentration, which is particularly advantageous for sustained delivery of bioactive compounds. The hydrogel loaded with a higher MP concentration (MP 400) exhibited a greater cumulative release (69.98%) compared to MP 200 (52%), as well as a higher zero‐order release rate constant (K₀ = 0.412 vs. 0.305).

This enhanced release can be attributed to the larger concentration gradient between the hydrogel matrix and the external medium in the LPC‐3AT‐MP 400 formulation, which increases the driving force for diffusion. Additionally, with a fixed crosslinker concentration, the matrix network in the LPC‐3AT‐MP 400 hydrogel may have become more saturated with the extract, leading to weaker binding interactions and facilitating a faster and more efficient release. Although the current system exhibited zero‐order behaviour, suggesting that the release is not purely diffusion‐controlled. The sustained and linear release profile implies a combination of diffusion and matrix relaxation, possibly indicating a non‐Fickian or anomalous transport mechanism. This is consistent with hydrogels that undergo swelling and structural relaxation during release. Given that the release reached a steady state prior to hydrogel disintegration (at the swelling equilibrium point), it can be inferred that the MP extract release was governed by controlled matrix‐driven mechanisms rather than simple Fickian diffusion.

### 4.12 Drug Encapsulation Efficiency

**FIGURE 10:**
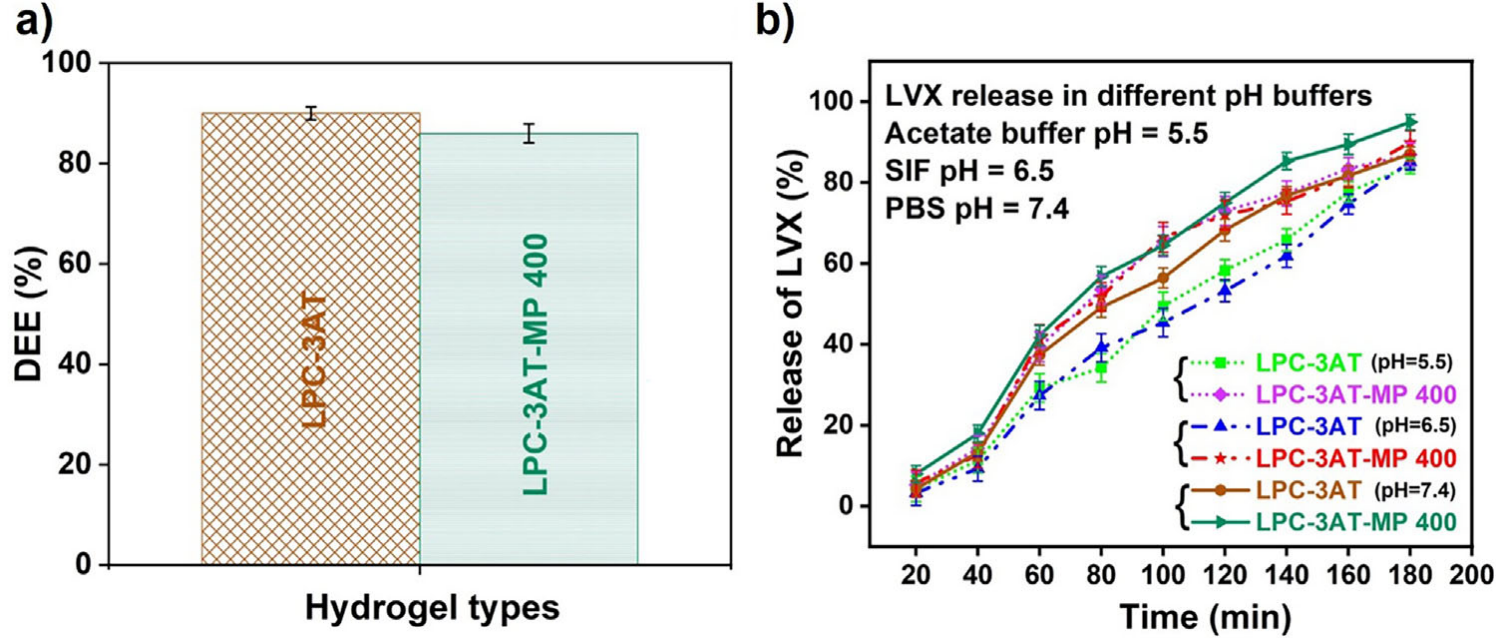
Drug encapsulation efficiency (DEE%) of levofloxacin (LVX)‐loaded hydrogels: LPC‐3AT and LPC‐3AT‐MP400, in PBS (pH 7.4). b) Levofloxacin (LVX) release profiles from LPC‐3AT and LPC‐3AT‐MP400 hydrogels in different pH buffers: acetate buffer (pH 5.5), simulated intestinal fluid (SIF, pH 6.5), and PBS (pH 7.4).

Drug encapsulation efficiency (DEE) is a critical parameter in hydrogel‐based drug delivery systems, reflecting the proportion of drug successfully entrapped within the polymer matrix. As shown in Figure 10a, the DEE of LPC‐3AT hydrogel was found to be approximately 89%, indicating a high encapsulation capacity. However, upon the incorporation of MP 400 plant extract, the DEE slightly decreased to 85% in the LPC‐3AT‐MP 400 formulation. This slight reduction in DEE may be attributed to the interaction of phytoconstituents present in the plant extract with the hydrogel network, which could interfere with optimal drug entrapment. The addition of plant extract might introduce additional hydrophilic or bulky groups, potentially disturbing the polymer‐drug interactions or modifying the network density, thereby slightly reducing the encapsulation efficiency. Despite the marginal decline, both formulations maintained high DEE values, suggesting their potential applicability in controlled drug delivery systems [46].

### 4.13 Levofloxacin Release Profile at Different pH Buffers

The release behaviour of levofloxacin from hydrogel matrices is primarily governed by drug diffusion through the water‐filled pores of the hydrogel, a process significantly influenced by the swelling capacity of the polymer network. In this study, the release profiles of levofloxacin were evaluated using two hydrogel formulations—LPC‐3AT and LPC‐3AT‐MP400—under different pH conditions that simulate physiological and pathological environments. The release was conducted at 37°C in acetate buffer (pH 5.5), simulated intestinal fluid (SIF, pH 6.5), and phosphate‐buffered saline (PBS, pH 7.4), as presented in Figure 10b.

Both formulations exhibited sustained and substantial levofloxacin release over 180 min, meeting the USP XXIV specification [47], for controlled‐release formulations, which requires more than 80% drug release within 3 h. Specifically, LPC‐3AT released 84% and 85% of levofloxacin at pH 5.5 and 6.5, respectively, while LPC‐3AT‐MP400 showed slightly enhanced release percentages of 86% and 89% under the same conditions. In PBS (pH 7.4), the cumulative release reached 87% for LPC‐3AT and 94% for LPC‐3AT‐MP400 within the same timeframe. Despite minor numerical differences, the release curves across all pH conditions largely overlapped, indicating that the hydrogel maintained effective drug delivery performance across a range of physiological pH values. The comparatively higher drug release observed in the MP400‐loaded hydrogel may be attributed to the presence of MP plant extract, which appears to slightly alter the hydrogel's structural properties. While the crosslinker concentration remained constant, the incorporation of MP likely weakened the internal network due to its bioactive constituents. These components may disrupt intra‐matrix interactions or reduce polymer–drug binding affinity, thereby facilitating greater water uptake and enhancing drug diffusion.

Importantly, drug release studies were conducted only up to the hydrogel's equilibrium swelling time (180 min). Beyond this point, the hydrogel network exhibited physical instability, including cracking, tearing, or fragmentation, which led to uncontrolled burst release and prevented accurate results assessment. Thus, only data within the stable swelling phase were considered. The diffusion exponent (n) value for both formulations was calculated to be 0.62, indicating a non‐Fickian (anomalous) transport mechanism. This suggests that drug release was influenced by a combination of Fickian diffusion and polymer matrix relaxation, possibly due to interactions between levofloxacin and the polymer or MP components. Overall, the results confirm the capability of both hydrogels to release levofloxacin in a controlled manner across a range of pH environments, with MP‐loaded formulations offering enhanced release performance likely due to modifications in matrix dynamics [7, 8, 35].

## 5 Conclusions

In this study, a novel, pH‐sensitive, biodegradable, eco‐friendly, and highly swellable ternary hydrogel systems based on locust bean gum, PVP, and chitosan were successfully fabricated using APTES crosslinker and infused with varying concentrations of *Mallotus philippensis* (MP) extract via a green synthesis approach, for controlled levofloxacin release. Studied the effect of MP concentration on LVX release profile. FT‐IR confirmed functional group interactions; SEM revealed a highly porous morphology; and TGA showed enhanced thermal stability, with LPC‐3AT‐MP 400 being the most stable. Swelling decreased with crosslinker addition (108 g/g at 180 min for LPC‐3AT), but increased with MP infusion (151 g/g at 120 min for LPC‐3AT‐MP 200). In different pH buffers, all LPCs showed minimal swelling at neutral and physiological pH, whereas, in electrolyte solutions, higher swelling was noticed in NaCl vs. CaCl₂, and delayed degradation in crosslinked samples, with LPC‐3AT showing 87.67% crosslinking density. Contact angle and porosity confirmed hydrophilic, porous structures. The addition of MP enhanced the antibacterial activity of LPC‐based hydrogels, demonstrating stronger inhibition against E. coli and S. aureus. Among the formulations, LPC‐3AT‐MP 200 and LPC‐3AT‐MP 400 exhibited the highest cytocompatibility. Among the kinetic models applied to the release of MP plant extract, the zero‐order model best described the release behaviour, demonstrated by high correlation coefficients (R^2^ = 0.98 for LPC‐3AT‐MP 200 and 0.99 for LPC‐3AT‐MP 400. The drug encapsulation efficiency (DEE) of LPC‐3AT was highest at 89%, but decreased to 85% upon MP extract addition, likely due to interactions between phytoconstituents and the hydrogel network, causing changes in network density. Drug release profiling at pH 5.5, 6.5, and 7.4 demonstrated that the hydrogel consistently maintained effective delivery performance, achieving over 80% drug release within 3 h in accordance with USP pharmacopeia standards. LPC‐3AT and LPC‐3AT‐MP 400 exhibited drug release of 87.04% and 94.5%, respectively, in PBS over 180 min. Despite minor variations, the release profiles were largely similar across all pH values, with a diffusion exponent (n = 0.62) indicating a non‐Fickian (anomalous) transport mechanism. One limitation of this study is the lack of application of various drug release kinetic models, the use of different plant extracts and drug models, as well as the absence of in vivo evaluation, which would provide a deeper understanding of the hydrogel's therapeutic potential.

## 6 Future Perspective

Future research should focus on detailed in vivo studies to evaluate the therapeutic efficacy, biocompatibility, and long‐term stability of the developed hydrogels. Additionally, exploring the use of different plant extracts, varying crosslinking densities, and other drug models could further optimize the hydrogels for diverse biomedical applications, such as localized drug delivery and wound healing.

## Conflicts of Interest

The authors declare no conflicts of interest.

## Data Availability

The data that support the findings of this study are available from the corresponding author upon reasonable request.
